# Pharmacological Induction of Transforming Growth Factor-Beta1 in Rat Models Enhances Radiation Injury in the Intestine and the Heart

**DOI:** 10.1371/journal.pone.0070479

**Published:** 2013-07-25

**Authors:** Marjan Boerma, Junru Wang, Vijayalakshmi Sridharan, Jean-Marc Herbert, Martin Hauer-Jensen

**Affiliations:** 1 Division of Radiation Health, Department of Pharmaceutical Sciences, University of Arkansas for Medical Sciences, Little Rock, Arkansas, United States of America; 2 Sanofi-Aventis, Paris, France; 3 Surgical Service, Central Arkansas Veterans Healthcare System, Little Rock, Arkansas, United States of America; Northwestern University Feinberg School of Medicine, United States of America

## Abstract

Radiation therapy in the treatment of cancer is dose limited by radiation injury in normal tissues such as the intestine and the heart. To identify the mechanistic involvement of transforming growth factor-beta 1 (TGF-β1) in intestinal and cardiac radiation injury, we studied the influence of pharmacological induction of TGF-β1 with xaliproden (SR 57746A) in rat models of radiation enteropathy and radiation-induced heart disease (RIHD). Because it was uncertain to what extent TGF-β induction may enhance radiation injury in heart and intestine, animals were exposed to irradiation schedules that cause mild to moderate (acute) radiation injury. In the radiation enteropathy model, male Sprague-Dawley rats received local irradiation of a 4-cm loop of rat ileum with 7 once-daily fractions of 5.6 Gy, and intestinal injury was assessed at 2 weeks and 12 weeks after irradiation. In the RIHD model, male Sprague-Dawley rats received local heart irradiation with a single dose of 18 Gy and were followed for 6 months after irradiation. Rats were treated orally with xaliproden starting 3 days before irradiation until the end of the experiments. Treatment with xaliproden increased circulating TGF-β1 levels by 300% and significantly induced expression of TGF-β1 and TGF-β1 target genes in the irradiated intestine and heart. Various radiation-induced structural changes in the intestine at 2 and 12 weeks were significantly enhanced with TGF-β1 induction. Similarly, in the RIHD model induction of TGF-β1 augmented radiation-induced changes in cardiac function and myocardial fibrosis. These results lend further support for the direct involvement of TGF-β1 in biological mechanisms of radiation-induced adverse remodeling in the intestine and the heart.

## Introduction

At least 50 percent of all cancer patients will receive radiotherapy at some stage of their cancer treatment. However, the extent to which radiotherapy can be used in cancer treatment is limited by side effects due to radiation exposure of non-cancer tissues that surround the treatment target. The intestine and the heart are dose-limiting organs in radiotherapy of the abdomen and the chest, respectively. Radiotherapy of abdominal and pelvic malignancies may lead to acute and chronic radiation enteropathy. Acute radiation enteropathy develops during radiotherapy as a result of intestinal crypt cell death, disruption of the epithelial barrier, and mucosal inflammation. Chronic radiation enteropathy may develop several years after radiotherapy and is characterized by progressive intestinal wall fibrosis and vascular sclerosis [1], [2]. Radiation-induced heart disease (RIHD) may present several years after radiotherapy of intrathoracic and chest wall tumors and involves accelerated atherosclerosis, conduction defects, and/or myocardial fibrosis [3]–[5]. Even though radiotherapy has been greatly improved over the last decades, radiation injury in organs such as the heart and intestine still limits the dose that can be delivered to the target. Hence, research is needed to identify biological mechanisms that underlie these toxicities.

Fibrosis is a prominent clinical manifestation of both radiation enteropathy and RIHD. Transforming Growth Factor-beta (TGF-β) is a multifunctional growth factor that plays an important role in fibrosis due to a variety of causes, including after radiation exposure [6]. Three mammalian isoforms of TGF-β exist, of which TGF-β1 is the prevalent form in most tissues. TGF-β1 is secreted by numerous cell types as part of a latent complex. Several factors including αvβ6 integrin, the mannose 6-phosphate/insulin-like growth factor II receptor, and proteases such as plasmin or mast cell chymases can dissociate TGF-β1 from its latent complex [7]–[9]. Ionizing radiation alone may also release TGF-β1 from the complex through the direct effect of reactive oxygen species [10]. Upon activation, TGF-β1 binds to a complex of type I and type II kinase receptors on many cell types. While in most cell types the type I receptor is Activin Receptor-Like Kinase 5 (ALK5), endothelial cells express an additional type I receptor, ALK1. The major intracellular signaling pathways induced by TGF-β1 involve proteins of the Smad family. Phosphorylated Smads form complexes with each other, whereupon they translocate into the nucleus and regulate gene expression [11], [12]. Through these pathways, TGF-β regulates many cellular functions, including the differentiation and activation of (myo)fibroblasts, the proliferation and permeability of endothelial cells, the transition of endothelial cells into fibroblasts, and the suppression of epithelial cell proliferation [13]–[16].

Xaliproden (SR 57746A) is an orally active non-peptide agonist of the serotonin 5-HT_1A_ receptor that was originally designed as a neuroprotective agent. Because not all clinical trials involving central nervous disorders showed positive outcomes, the development of xaliproden as a novel drug in the treatment of these disorders was discontinued [17]. However, functional characterization of rats after oral treatment with xaliproden revealed that it markedly enhanced circulating levels of TGF-β1 by mechanisms that were only partly understood, and that plasma levels remained increased for as long as the compound was administered (Herbert, personal communication). Hence, xaliproden may be used as a pharmacological tool to examine the mechanistic role of TGF-β1 in rat models of disease. To identify the role of TGF-β1 in intestinal and cardiac radiation injury, we studied the influence of its induction with xaliproden in rat models of radiation enteropathy and RIHD.

## Materials and Methods

### Ethics Statement

This study conformed to the Guide for the Care and Use of Laboratory Animals of the National Institutes of Health. The protocol was approved by the University of Arkansas for Medical Sciences’ Institutional Animal Care and Use Committee.

### Animals

Male Sprague-Dawley rats (180–200 g) were obtained from Harlan Laboratories (Indianapolis, IN). All animals were housed 2–3 per cage in our Division of Laboratory Medicine on a 12∶12 light-to-dark cycle with free access to food and water. Rats were either used for the radiation enteropathy model or the model of RIHD. Because it was uncertain to what extent TGF-β induction may enhance radiation injury in heart and intestine, animals were exposed to irradiation schedules that cause mild to moderate radiation injury in our animal models. Where a single dose of 18 Gy causes moderate cardiac radiation injury in the rat, it would cause extensive acute radiation injury in the intestine. We therefore selected a fractionated protocol for the radiation enteropathy model of 7 daily fractions of 5.6 Gy.

### Radiation Enteropathy Model

A surgical model for localized small intestine irradiation was prepared as described previously [18]. Briefly, rats were fasted overnight, anesthetized, and orchiectomized. A loop of ileum was sutured to the inside of the scrotum. The model creates a scrotal hernia that contains a 4-cm loop of small intestine that can be irradiated locally without significant radiation exposure of other tissues, while the intestine remains functional and within the abdominal cavity. The model minimizes manipulation during irradiation and produces radiation-induced changes similar to those seen clinically. The surgical procedure itself does not cause structural, functional, or cellular changes in the intestine.

Two weeks after the scrotal hernia preparation, the rats were anesthetized with 2.5% isoflurane, placed in the supine position, and the transposed bowel segment within the scrotal hernia was irradiated with 7 once-daily fractions of 5.6 Gy with a Seifert Isovolt 320 X-ray machine (Seifert X-Ray Corporation, Fairview Village, PA) operated at 250 kV and 15 mA with 3 mm aluminum filtration, at a dose rate of 4.49 Gy/min. Dosimetry has been described before [19]. Rats were observed for 2 weeks or 12 weeks after localized intestine irradiation to examine either early or chronic manifestations of radiation enteropathy.

### Model of Localized Heart Irradiation

A separate cohort of rats was used for our RIHD studies, as described before [20]. After two weeks of acclimatization, rats were anesthetized with 2.5% isoflurane and irradiated with a Seifert Isovolt 320 X-ray machine (Seifert X-Ray Corporation) operated at 250 kV and 15 mA with 3 mm aluminum and 1.85 mm copper filtration, at a dose rate of 1.17 Gy/min. Radiation was delivered locally on the heart using parallel opposed fields (anterior-posterior 1∶1) with a diameter of 19 mm, while the rest of the animal was shielded with lead plates. Rats were observed for 6 months after irradiation to examine chronic manifestations of cardiac radiation injury.

### Xaliproden Administration

Rats in the RIHD model were randomly divided into 4 groups (Table 1), and rats in the radiation enteropathy model were randomly divided into 8 groups (Table 2), to administer xaliproden or vehicle in combination with local irradiation or sham-irradiation. Xaliproden was kindly provided by Sanofi-Aventis (Bridgewater, NJ). In both radiation models, rats were treated with xaliproden at a dose of 10 mg/kg body weight per day from 3 days before irradiation until the endpoint of the studies. To ascertain a fast rise of plasma TGF-β levels in response to xaliproden, and have elevated TGF-β levels at the time of irradiation, for the first 3 weeks of the experiments, xaliproden was administered by oral gavage. To avoid prolonged repeated handling of animals, after the first 3 weeks of gavage, oral administration of xaliproden was performed by adding it to the standard rodent chow Teklad TD8640 (Harlan), at 125 mg xaliproden/kg chow. Weekly chow intake was monitored per cage throughout the experiments to verify xaliproden dosage.

**Table 1:** Body weights (average ± SEM) in the radiation enteropathy model.

Radiation	Vehicle/xaliproden	N	Time after irradiation (weeks)	N at endpoint	Body Weight (g)
7×0 Gy	Vehicle	5	2	5	330.4±8.4
7×0 Gy	Xaliproden	15	2	15	302.7±6.7*
7×5.6 Gy	Vehicle	13	2	13	296.5±3.1#
7×5.6 Gy	Xaliproden	15	2	15	261.9±9.5*
7×0 Gy	Vehicle	7	12	7	390.3±10.2
7×0 Gy	Xaliproden	7	12	7	372.4±11.2*
7×5.6 Gy	Vehicle	13	12	13	419.6±5.5
7×5.6 Gy	Xaliproden	14	12	8	348.0±14.9*

* Significant difference with vehicle-treated animals (p<0.01).

# Significant difference with sham-irradiated animals (p<0.05).

**Table 2:** Body weights, heart weights, and heart/body weight ratios (average ± SEM) in the RIHD model.

Radiation	Vehicle/xaliproden	N	Time afterirradiation (weeks)	Body Weight (g)	Heart Weight (g)	Heart/Body Weight
0 Gy	Vehicle	6	24	539.0±24.4	1.6±0.1	3.1±0.1
0 Gy	Xaliproden	7	24	447.1±10.1*	1.4±0.1*	3.0±0.1
18 Gy	Vehicle	10	24	513.4±13.6	1.6±0.04	3.1±0.1
18 Gy	Xaliproden	10	24	388.9±7.7*	1.2±0.03*	3.2±0.1

* Significant difference with vehicle-treated animals (p<0.01).

### Plasma and Tissue TGF-β Measurements

A separate group of rats was used to determine plasma levels of TGF at 1, 6, and 24 hours after oral delivery of xaliproden by gavage. Peripheral venous blood samples (1 ml) were obtained from the tail vein with EDTA as an anticoagulant and were centrifuged at 1,000 g for 20 min. The plasma was isolated and stored at –70°C until the assay was performed. To activate latent TGF-ß1 into immunoreactive TGF-ß1 detectable by the immunoassay test, an acidification (by adding 100 µl 2.5 N acetic acid/10 M urea to every 100 µl sample) and neutralization (by adding 100 µl 2.7 N NaOH/1 M HEPES) (pH 7.2–7.6) were performed. Plasma and tissue levels of TGF-α, TGF-β1, and TGF-β2 were measured with conventional ELISAs.

### Cardiac in vivo and ex vivo Function Measurements

Echocardiographic parameters were obtained at 3 months and 6 months after irradiation with an HP Sonos 5500 system (Philips Medical Systems, Eindhoven, The Netherlands) and S12 probe (5–12 MHz), as described before [20]. Hair was removed from the chest with clippers and parasternal short axis B-mode recordings at the mid left ventricular level were obtained. Fractional area change was calculated as: (left ventricular diastolic area –left ventricular systolic area)/left ventricular diastolic area.

Langendorff studies were performed to determine *ex vivo* cardiac function at 6 months after local heart irradiation. Hearts were isolated from rats in each of the treatment groups and immediately perfused via the aorta with an oxygenated Krebs-Henseleit solution (37°C) at a flow rate of 8 ml/g heart/min. Both atria were removed and the ventricles were paced with platinum contact electrodes positioned on the interventricular septum to obtain a heart rate of 250 beats/min. A fluid-filled balloon catheter was placed in the left ventricle and left ventricular diastolic and systolic pressures were measured at various balloon volumes between 80 µl and 300 µl (a range that elicited maximum contractility in all preparations). Coronary pressure was monitored continuously with a pressure transducer (model PT300, Grass Technologies, West Warwick RI). All data were digitized and analyzed with *CODAS* acquisition and analysis software (DataQ Instruments, Akron, Ohio, USA). After Langendorff studies the hearts were weighed and processed for histology and immunohistochemistry.

According to a method of Radke et al [21], pressure data obtained from Langendorff preparations were converted into wall stress to correct for possible differences in left ventricular geometry. Left ventricular wall stress was calculated as: Left ventricular pressure/[(left ventricular wall volume/balloon volume +1)2/3 −1]. Left ventricular wall volume was calculated as: heart weight/1.05, assuming that the heart weight mainly reflected left ventricular weight.

### Quantitative Histopathology and Morphometry

Specimens of (sham)-irradiated intestine and heart were cut longitudinally, fixed in methanol Carnoy’s solution (60% methanol, 30% chloroform, 10% acetic acid) and embedded in paraffin. Sections of 5 µm were used for histopathology, morphometry, and immunohistochemistry.

Heart sections were incubated in Picrosirius red stain (American MasterTech, Lodi, CA) supplemented with Fast Green (0.01% w/v, Fisher Scientific, Pittsburgh, PA) for 2 hours. Quantitative assessment of the Picrosirius red/Fast Green staining was performed with computerized image analysis (Image-Pro Plus, Media Cybernetics, Silver Spring, MD). The relative area of collagens was calculated as the Picrosirius red-stained area/area stained by Picrosirus red+Fast Green×100%.

Sections of intestine were stained with Hematoxylin & Eosin and used to determine radiation injury score, mucosal surface area, and intestinal wall thickness as described before [22], [23]. The radiation injury score provides a global measure of the severity of structural radiation injury in the intestine. It is a composite histopathologic scoring system that has been extensively used and validated in our laboratory. Histopathologic parameters of radiation injury (mucosal ulcerations, epithelial atypia, thickening of subserosa, vascular sclerosis, intestinal wall fibrosis, ileitis cystica profunda, and lymph congestion) were assessed and graded from 0 to 3. The sum of the scores for the individual alterations constitutes the radiation injury score. All specimens were evaluated in a blinded fashion by two separate researchers, and discrepancies in scores were resolved by consensus.

A radiation-induced decrease in surface area of the mucosa is a sensitive parameter of intestinal radiation injury. Mucosal surface area was measured in vertical sections using a stereologic projection/cycloid method as described by Baddeley et al [24] and adapted by us to our model system [25]. This method does not require assumptions about the shape or orientation of the specimens and thus circumvents problems associated with most other procedures for surface area measurement.

Intestinal wall thickening is a measure of both reactive intestinal wall fibrosis and intestinal smooth muscle cell hyperplasia. In contrast, subserosal thickening reflects mainly reactive fibrosis. Intestinal wall thickness and subserosal thickness were measured with computer-assisted image analysis (Image-Pro Plus, Media Cybernetics). All measurements were made by utilizing a 10× objective lens. A total of 5 areas, 500 µm apart, were selected for measurement, with 3 measurements taken per area. The average of all 5 areas was used as a single value for statistical calculations.

### Immunohistochemistry

For determination of myofibroblast numbers in the heart, sections were immunostained for α-smooth muscle cell (SMC) actin. Endogenous peroxidase was blocked with 1% H_2_O_2_ in methanol. Non-specific antibody binding was reduced by TBS containing 10% normal goat serum (Vector Laboratories, Burlingame, CA) or 10% normal donkey serum (Jackson ImmunoResearch, West Grove, PA), 3% dry powdered milk and 0.2% BSA. Sections were incubated with rabbit anti α-SMC actin (Abcam, Cambridge, MA) for 2 hours at 1∶200, followed by either biotinylated goat anti-rabbit IgG (Vector Laboratories), or Alexa Fluor 594-labeled donkey anti-rabbit IgG (Invitrogen, Carlsbad, CA), each for 30 minutes at 1∶400. Biotinylated secondary antibodies were visualized with an avidin-biotin-peroxidase complex (Vector Laboratories) followed by 0.5 mg/ml 3,3-diaminobenzidine tetrahydrochloride (DAB, Sigma-Aldrich) and 0.003% H_2_O_2_ in TBS. Hematoxylin was used as a counterstain. Cardiac areas immunoreactive for myofibroblasts were scored blindly on a graded scale as follows: 0 (no myofibroblasts), 1 (myofibroblasts covered less than 5 optical areas at a 40× magnification), 2 (myofibroblasts covered 5 or more optical areas at a 40× magnification).

Sections of small intestine were immunostained for myeloperoxidase (MPO), extracellular matrix-associated TGF-β, and collagen types I and III. Endogenous peroxidase was blocked with 1% H_2_O_2_ in methanol for 30 minutes at room temperature. Non-specific antibody binding was reduced by 10% normal rabbit serum or 10% normal goat serum (Vector Laboratories) in 3% dry powdered milk in TBS for 30 min. Sections were then incubated with rabbit anti-MPO (Dako, Glostrup, Denmark) at 1∶100, pan-specific rabbit anti-TGF-β antibody (R&D, Minneapolis, MN) at 1∶300, goat anti-collagen I, or goat anti-collagen III (Southern Biotechnology Associates, Birmingham, AL) each at 1∶100, for 2 hours at room temperature. This was followed by a 30 min-incubation with biotinylated goat anti-rabbit IgG or biotinylated rabbit anti-goat IgG (Vector Laboratories), each at 1∶400. Sections were incubated with pre-formed avidin-biotin-peroxidase complex (Vector Laboratories) for 30 minutes and visualized with 0.5 mg/ml DAB (Sigma-Aldrich) and 0.003% H_2_O_2_ in TBS. Hematoxylin was used as a counterstain. The number of MPO-positive cells was determined by counting in 20 field (40× magnification), selected according to a predetermined grid pattern. Quantitative assessment of TGF-β and collagen immunoreactive areas was performed with computerized image analysis using the software Image-Pro Plus (Media Cybernetics).

### RNA Isolation and Real-time PCR

Specimens of (sham)-irradiated intestine and heart were snap-frozen in liquid nitrogen and stored at −80°C. Total RNA was isolated with Ultraspec™ RNA reagent (Biotecx Laboratories, Houston, TX), using a motorized homogenizer. After treatment with RQ-DNAse I (Promega, Madison, WI) at 37°C for 30 min, cDNA was synthesized using the High Capacity cDNA Archive Kit™ (Applied Biosystems, Foster City, CA). Steady-state mRNA levels were measured with real-time quantitative PCR (TaqMan™) using either the ABI Prism 7700 Sequence Detection System or the ABI Prism 7500 Fast system, TaqMan Universal PCR mastermix, and the following pre-designed TaqMan Gene Expression Assays™ for rat: TGF-β1 (Rn00572010_m1), endoglin (Rn01438763_m1), plasminogen activator inhibitor-1 (PAI-1, Rn00561717_m1), and Inhibitor of DNA binding-1 (Id-1, Rn00562985_s1). Relative mRNA levels were calculated with the ΔΔCt method, using eukaryotic 18S rRNA (Hs99999901_s1) or rat GAPDH (Rn01775763_g1) as internal control (all Applied Biosystems).

### Statistical Analysis

Data were evaluated with the software packages NCSS 2007 (NCSS, Kaysville, UT) and StatXact 5 (Cytel Software, Cambridge, MA). Repeated measures ANOVA was used to test data from echocardiography and Langendorff. Frequencies of scores for myofibroblasts were examined with a Chi-Square test. Gene expression data were tested with the non-parametric Mann-Whitney test. All other data were tested with two-way ANOVA followed by the Newman-Keul’s multiple range test. The criterion for significance was p<0.05. Data are reported as average ± standard error of the mean (SEM).

## Results

### Xaliproden Increased Plasma TGF-β1 Levels

**Figure 1:**
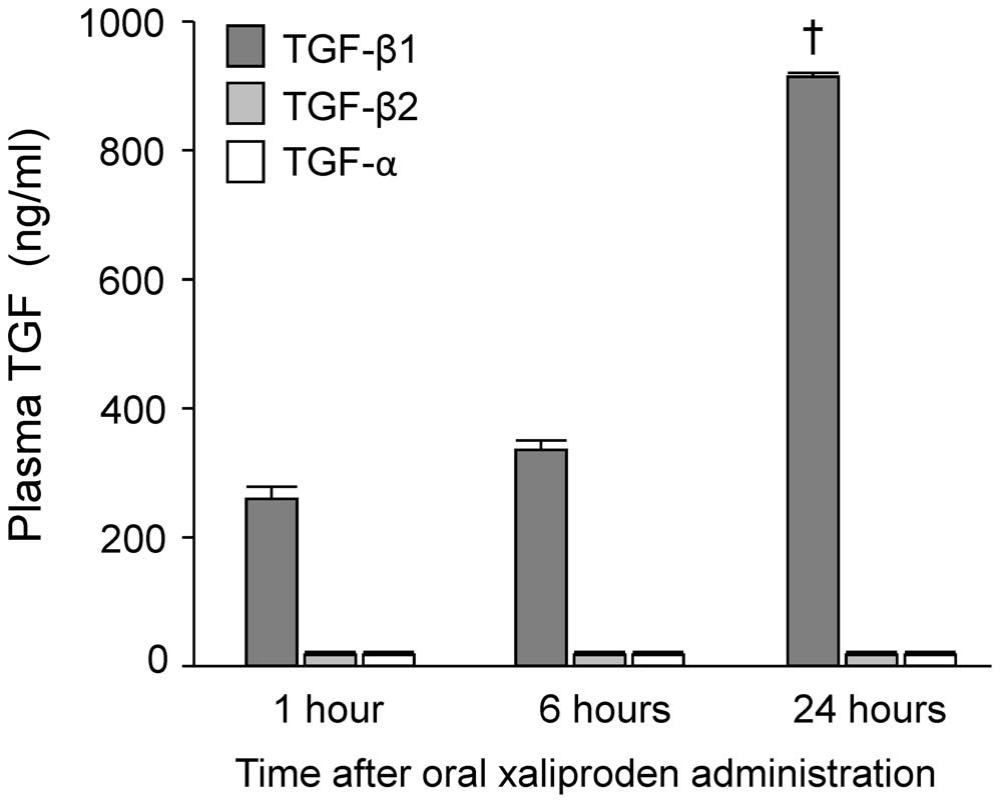
Effects of xaliproden on plasma TGF levels in unirradiated animals. Xaliproden caused a significant increase in plasma levels of latent TGF-β1, but not of TGF-α or TGF-β2 at 24 hours after oral administration. Average ± SEM, n = 10. ^†^p<0.0001.

The effects of oral administration of xaliproden on plasma and tissue protein levels of TGF-β were examined. Xaliproden significantly enhanced circulating TGF-β1 levels, but did not alter levels of TGF-α or TGF-β2 at 24 hours after administration (Figure 1). The increase in TGF-β1 levels seemed to occur only in the plasma, as TGF-β1 protein did not change in skeletal muscle, cardiac left ventricle, cardiac atria, liver, kidney or small intestine (data not shown).

### Xaliproden Enhanced TGF-β1 Signaling in the Irradiated Intestine and Heart

**Figure 2:**
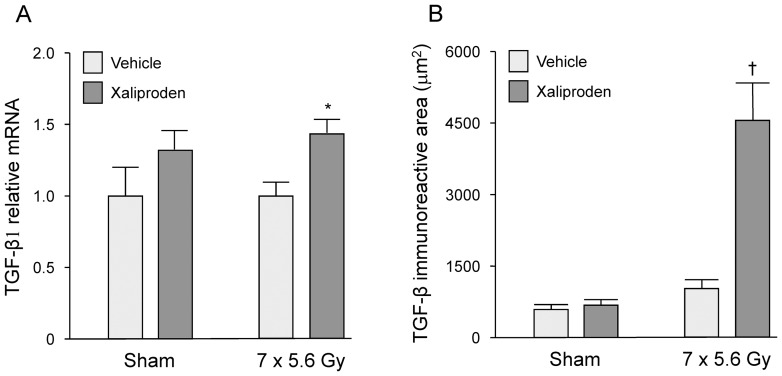
Effects of radiation and xaliproden on intestinal TGF-β expression. A 2-weeks administration of xaliproden significantly enhanced relative mRNA levels of TGF-β1 (A) and increased TGF-β immunoreactivity (B) in the irradiated small intestine. Average ± SEM, n = 5 (sham-irradiated) or 13–15 (irradiated). *p<0.05, ^†^p<0.0001. Representative micrographs of TGF-β immunohistochemical stainings are shown in Figure S1.

TGF-β1 is known to upregulate its own expression via a positive feedback loop. Hence, a 2-weeks administration of xaliproden caused a significant increase in TGF-β1 relative mRNA levels and TGF-β immunoreactive area in the irradiated small intestine (Figure 2, Figure S1).

**Figure 3:**
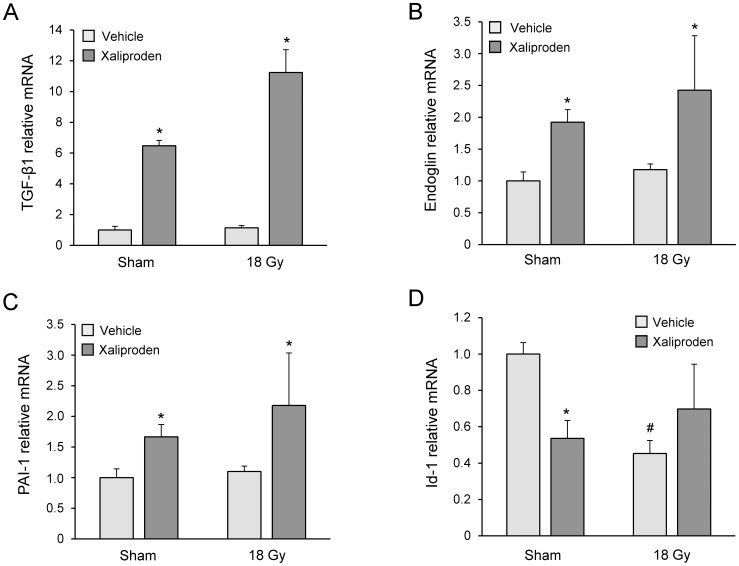
Effects of radiation and xaliproden on left ventricular gene expression of TGF-β1 and TGF-β pathway mediators. Xaliproden caused a significant increase in left ventricular mRNA of TGF-β1 (A), endoglin (B), PAI-1 (C), and both radiation and xaliproden caused a downregulation of Id-1 mRNA when compared to vehicle-treated rats (D). Average ± SEM, n = 3. ^#^Significant difference with sham-irradiated animals (p<0.05), *Significant difference with vehicle-treated animals (p<0.05).

In the heart, a 6-months treatment with xaliproden significantly enhanced left ventricular gene expression of TGF-β1 (Figure 3A). Treatment with xaliproden also enhanced the expression of endoglin, a co-receptor that modulates the balance between the TGF-β receptors ALK1 and ALK5 in endothelial cells (Figure 3B), and enhanced the expression of PAI-1, a transcriptional target of the ALK5 pathway (Figure 3C). Both local heart irradiation and xaliproden caused a downregulation of Id-1 mRNA, a transcriptional target of the ALK1 pathway (Figure 3D).

### Xaliproden Altered Heart and Body Weights

In the radiation enteropathy model, all of the vehicle-treated irradiated rats survived until the endpoints of the experiments (2 weeks or 12 weeks after irradiation). A total of 29 animals received local intestine irradiation in combination with xaliproden. Six of these animals were moribund and hence had to be sacrificed between 1 and 11 weeks after irradiation. The exact cause of their moribund state was unknown. Table 1 shows numbers of animals remaining in each treatment group. Among the remaining animals, at 2 weeks after irradiation a reduction in body weight was observed. Xaliproden caused a reduction in body weight in both sham-irradiated and intestinal irradiated rats at 2 weeks and at 12 weeks (Table 1).

In the local heart irradiation studies, all animals survived up to the 6-month time point. A 6-months treatment with xaliproden caused significant reductions in body weight and heart weight in both sham-irradiated animals and irradiated animals (Table 2).

### Xaliproden Aggravated Some Effects of Radiation on Cardiac Function

**Figure 4:**
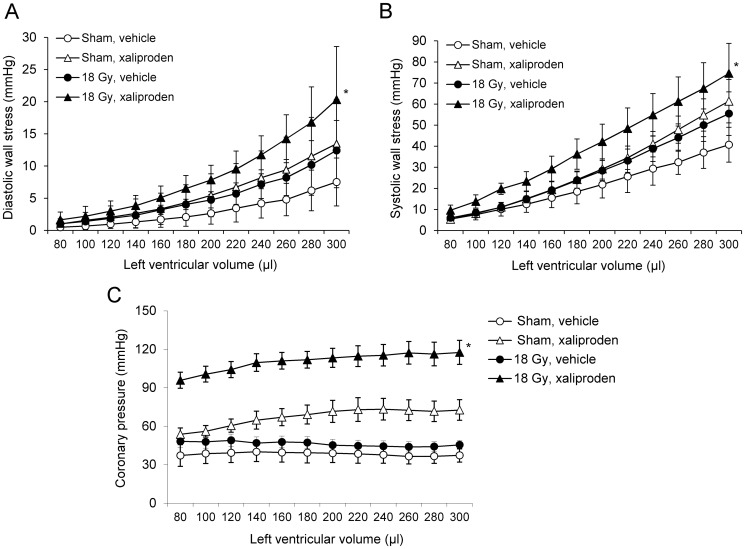
*Ex vivo* cardiac function analysis at 6 months after local heart irradiation and xaliproden treatment. Xaliproden significantly enhanced the effects of radiation on diastolic wall stress (A), systolic wall stress (B), and coronary pressure (C) as measured in Langendorff isolated perfused hearts at 6 months after local heart irradiation. Average ± SEM, n = 3–6. *Significant difference with sham-irradiated vehicle-treated animals (p<0.05).

The effects of local heart irradiation and xaliproden on cardiac function were measured with echocardiography at 3 and 6 months after irradiation (Table 3), and with Langendorff isolated perfused heart preparations at 6 months after irradiation (Figure 4). At 3 months after a single dose of 18 Gy, significant decreases were found in left ventricular inner diameter (LVID) in diastole, and in left ventricular systolic and diastolic area. Xaliproden augmented the reduction in the diastolic LVID in irradiated animals. No changes were found in fractional area change, (Table 3), fractional shortening, or thickness of left ventricular walls or the intraventricular septum (data not shown). None of the *in vivo* parameters were significantly altered at 6 months after 18 Gy. On the other hand, when hearts were isolated at this time and immediately perfused with the Langendorff method, hearts from animals treated with xaliproden alone seemed to display increased left ventricular diastolic wall stress, systolic wall stress, and coronary pressure, although these effects were not statistically significant, while hearts from animals treated with radiation in combination with xaliproden showed a significant increase in these parameters (Figure 4).

**Table 3:** Echocardiographic parameters (average ± SEM) at 3 and 6 months after local heart irradiation.

Radiation	Vehicle/xaliproden	Time afterirradiation(months)	N	LVID inDiastole(mm)	Left VentricularArea in Systole(mm^2^)	Left VentricularArea in Diastole(mm^2^)	FractionalArea Change(%)
0 Gy	Vehicle	3	6	9.2±0.2	6.7±0.4	3.3±0.4	51.5±3.2
		6	6	8.7±0.2	6.0±0.2	2.7±0.4	55.5±6.3
0 Gy	Xaliproden	3	6	8.8±0.3	6.1±0.3	2.8±0.2	54.0±2.8
		6	6	8.6±0.2	5.6±0.2	2.7±0.2	51.2±2.6
18 Gy	Vehicle	3	10	8.7±0.1#	5.8±0.2#	2.5±0.1#	56.6±1.7
		6	10	8.4±0.3	5.7±0.3	2.3±0.2	59.6±2.8
18 Gy	Xaliproden	3	10	8.1±0.3*	5.2±0.3	2.4±0.1	54.8±2.7
		6	10	8.0±0.2	5.1±0.3	2.2±0.2	56.7±1.8

# Significant difference with sham-irradiated animals (p<0.05).

* Significant difference with vehicle-treated animals (p<0.05).

### Xaliproden Aggravated Histopathological Manifestations of RIHD

**Figure 5:**
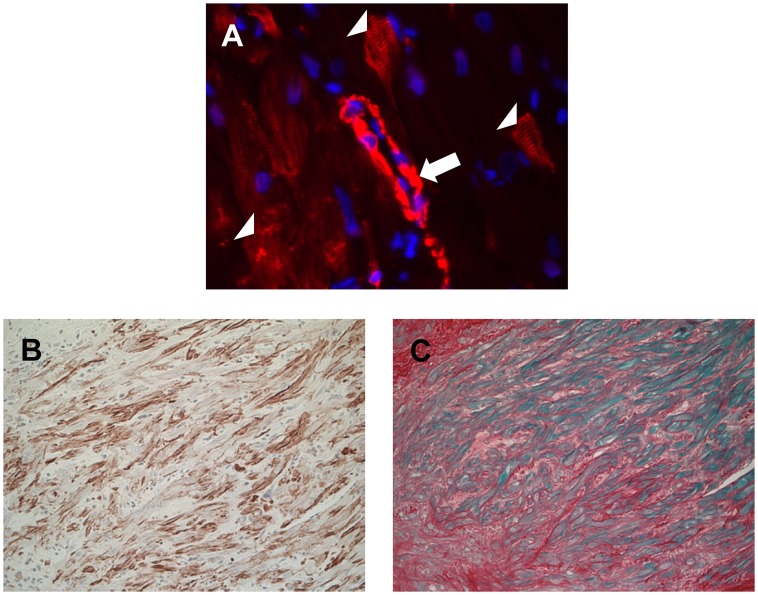
Immunohistochemical evidence of myofibroblasts after local heart irradiation. Immunohistochemistry with anti α-SMC antibodies revealed vascular smooth muscle cells with positive staining (arrow) and spindle shaped non-vascular cells with cross-striational α-SMC staining (myofibroblasts, arrowheads) (A). These myofibroblasts were detected in irradiated hearts only, in subendocardial areas near the cardiac valves (B). These areas showed severe fibrosis in the Picrosirius Red+Fast Green staining (C).

To detect myofibroblasts, heart sections were stained with antibodies against α-SMC actin. In addition to vascular SMC, the antibody staining revealed non-vascular cells with a spindle shape and a cross-striational staining pattern for α-SMC actin (Figures 5A and 5B), both characteristics of differentiated myofibroblasts. These myofibroblasts were detected only in irradiated hearts, in fibrotic subendocardial areas near the cardiac valves (Figure 5C) and in some areas of myocardial degeneration. The number of myofibroblasts in each section was scored blindly on a scale from 0 to 2. Xaliproden caused a significant increase in the number of myofibroblasts in irradiated hearts (Table 4). In addition, a significant increase in myocardial collagen area was observed at 6 months after irradiation. A trend of increased collagen deposition was observed in both sham-irradiated and in irradiated animals treated with xaliproden, but the increase was not statistically significant (Table 4, Figure S2).

**Table 4:** Left ventricular collagen area (average ± SEM) and scores for α-SMC actin containing myofibroblasts at 6 months after local heart irradiation.

Radiation	Modifier	N	Collagenarea (%)	Myofibroblastscore (0–2)
0 Gy	Vehicle	3	6.8±1.2	0,0,0
0 Gy	Xaliproden	4	9.6±0.7	0,0,0,1
18 Gy	Vehicle	7	11.4±1.2#	0,0,0,0,0,1,2
18 Gy	Xaliproden	7	12.4±1.3	1,1,1,1,2,2,2*

# Significant difference with sham-irradiated animals (p<0.05).

* Significant difference with all other groups (p<0.05).

Representative micrographs of histopathological stainings are shown in Figure S2.

### Xaliproden Enhanced Histopathological Manifestations of Radiation Enteropathy

**Figure 6:**
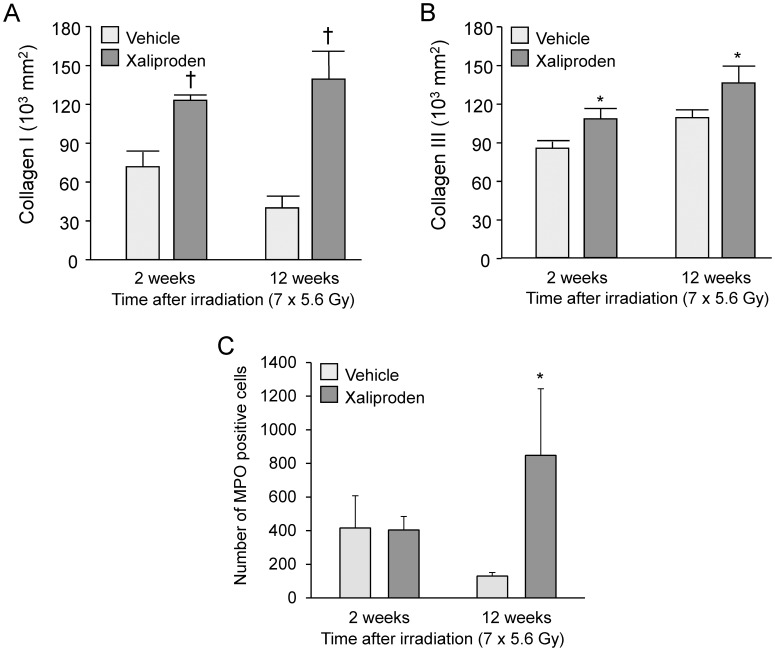
Histopathological manifestations of radiation enteropathy. Xaliproden significantly reduced mucosal surface area (A) at 12 weeks after local irradiation of the small intestine, and enhanced radiation injury score (B), intestinal wall thickness (C) and serosal thickness (D) at 2 weeks and 12 weeks after irradiation. Average ± SEM, n = 5 (sham-irradiated) or 13–15 (irradiated). *p<0.05, ^†^p<0.001. Representative micrographs of histopathological stainings are shown in Figure S3.

**Figure 7:**
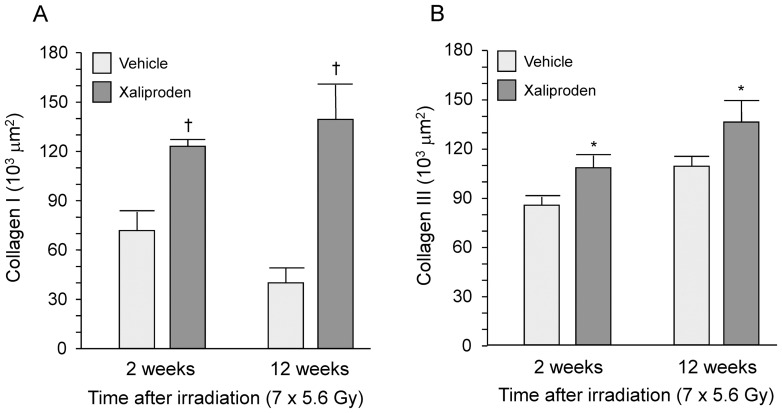
Immunohistochemical analysis of collagen deposition and MPO positive cells in radiation enteropathy. Xaliproden caused a significant increase in deposition of collagen types I (A) and III (B) at 2 weeks and at 12 weeks and a significant increase in the number of MPO positive cells (C) at 12 weeks after small intestine irradiation. Average ± SEM, n = 5–15. *p<0.05, ^†^p<0.001. Representative micrographs of immunohistochemical stainings are shown in Figure S4 and S5.

Representative micrographs of histopathological stainings are shown in Figure S3. In the radiation enteropathy model, xaliproden significantly reduced mucosal surface area in irradiated animals at 12 weeks (Figure 6A), and enhanced histopathological manifestations of radiation injury (Figure 6B), radiation-induced fibrotic thickening of the intestinal wall (Figure 6C) and subserosa (Figure 6D), at 2 weeks and 12 weeks after irradiation. In addition, xaliproden caused a significant deposition of collagen type I (Figure 7A) and collagen type III (Figure 7B, Figure S4) in the intestinal wall at both time points, and a significant increase in the number of MPO-positive cells (neutrophils) at 12 weeks after irradiation (Figure 7C, Figure S5).

## Discussion

TGF-β1 is considered a key growth factor in the development of radiation fibrosis. While most of the experimental data that provide proof for the role of TGF-β1 in radiation fibrosis are obtained from *in vitro* studies or from animal models that correlate TGF-β1 expression with normal tissue radiation injury [14], [26]–[28], some previous studies have addressed the role of TGF-β in radiation injury by inhibiting its expression or its signaling [29]–[32]. To our knowledge, this study is the first to use pharmacological induction of TGF-β1 to study its role in radiation injury. Plasma levels of TGF-β1 were increased by administering xaliproden to rats. Xaliproden is an agonist of the serotonin 5-HT_1A_ receptor, originally designed as a neuroprotective agent, but no longer in clinical trials [17]. Oral administration of xaliproden in rats causes an upregulation of circulating TGF-β1 levels by mechanisms that are not yet fully understood.

In our models of local intestine irradiation and local heart irradiation, xaliproden enhanced the number of myofibroblasts and aggravated radiation fibrosis and other histopathological and functional manifestations of radiation injury. We cannot exclude that xaliproden may have caused some of these effects via mechanisms not involving TGF-β1. The 5-HT_1A_ receptor is expressed on neurons and glial cells. Hence, xaliproden has been shown to enhance intestinal motility and luminal fluid content in rats [33]. However, these effects may be considered to reduce manifestations of radiation enteropathy in the rat, rather than aggravate them as we have observed in our studies. Xaliproden reduced inflammation and the expression of tumor necrosis factor-α and interferon-γ in a rat model of autoimmune encephalomyelitis [34]. However, reduced inflammation may again be considered to reduce manifestations of radiation injury in the intestine and the heart, rather than aggravate them. The worsening of manifestations of radiation injury in our rat models, together with severalfold up-regulations of circulating TGF-β1 and enhanced ALK5 signaling in response to xaliproden, suggest that worsening of radiation injury was caused primarily by increased TGF-β1.

Animal models of radiation enteropathy have shown an up-regulation of TGF-β in the intestinal wall [8], [35]–[38]. The current study shows that TGF-β1 enhances both acute and chronic radiation fibrosis and prevents regeneration of the intestinal mucosa. Indeed, TGF-β1 inhibits intestinal epithelial cell proliferation [15], [16], [39]. TGF-β also plays an important role in regulating the immune system in the intestinal wall [40]. We observed an increase in the number of MPO positive cells after TGF-β induction in late radiation injury. Although TGF-β1 is known for its pleiotropic immunosuppressive effects [41], enhanced intestinal wall fibrosis due to local overexpression of TGF-β1 is associated with enhanced inflammatory infiltration [42]. In congruence with the current study, inhibition of TGF-β with a soluble TGF-β type II receptor resulted in a reduction in the radiation injury score, enhanced mucosal surface area, and reduced intestinal wall fibrosis in a mouse model of radiation enteropathy [29]. Hence, inhibition of TGF-β1 signaling may reduce clinical manifestations of radiation enteropathy [43].

Myofibroblasts are considered the main cell type involved in fibrosis and wound healing, responsible for wound contraction and collagen deposition [44]. Myofibroblast differentiation and activation is thought to result from the combined action of mechanical tension and the extracellular presence of TGF-β and the ED-A splice variant of cellular fibronectin [45]. Indeed, in our model of RIHD, radiation-induced myofibroblasts were mainly found in the subendocardium, which is known to endure most mechanical tension within the myocardium [46], [47]. In addition, the subendocardium is more vulnerable to ischemia [48], which may make it more prone to cardiomyocyte degeneration and replacement fibrosis. We found an increased deposition of the ED-A splice variant of fibronectin in areas of myocardial radiation fibrosis. Xaliproden had no effect on ED-A fibronectin (data not shown), suggesting that its effects on myofibroblast numbers was mainly via the induction of TGF-β1.

The endothelial TGF-β receptor complex containing the type I receptor ALK1 signals via phosphorylation of Smad1/5, while activation of ALK5 leads to phosphorylation of Smad2/3. One of the direct transcriptional targets of the ALK1 pathway is Id-1, while PAI-1 expression is induced by the ALK5 pathway. The ALK1 and ALK5 pathways have opposite effects on endothelial cell functions [26], [49], and the balance between ALK1 and ALK5 is largely regulated by the co-receptor endoglin [50]. Hence, endoglin may regulate normal tissue radiation injury [51]. By using Id-1 and PAI-1 expression as readouts, previous studies have shown that ionizing radiation causes a long-term shift in the endothelial balance of TGF-β signaling, with reduced activation of the ALK1 pathway and enhanced signaling via ALK5 [26], [52], [53]. Our data suggest that reduced ALK1 signaling also occurs in the irradiated heart, while administration of xaliproden enhanced the ALK5 pathway. Although little is known about the consequences of an endothelial ALK1 to ALK5 shift in the heart, it may be a possible contributor to the long-term changes that occur in the microvasculature [54], [55] and the coronary arteries. While radiation injury in intima and media occur in rat models of local heart irradiation [56], radiation-induced accelerated atherosclerosis is not common in rats and, hence, was not observed in the current study. On the other hand, we found that local heart irradiation in combination with xaliproden caused an increase in coronary pressure, as measured in *ex vivo* perfused hearts at 6 months after irradiation. Indeed, TGF-β can promote arterial stiffness, through its effects on vascular endothelial cells, SMC and the extracellular matrix [57]–[59].

In conclusion, this study used a pharmacological approach to up-regulate TGF-β1 and provide evidence for the direct involvement of TGF-β1 in radiation-induced adverse remodeling in the small intestine and the heart.

## Supporting Information

Figure S1 **Representative micrographs of TGF-β immunostaining in the radiation enteropathy model.** TGF-β immunoreactivity at 12 weeks after sham-irradiation (A) and at 12 weeks after 7×5.6 Gy local intestine irradiation (B). 5× Magnification, scale bar: 400 µm.
(TIF)

Figure S2 **Representative micrographs of histological findings in the RIHD model.** Picrosirius red+Fast Green staining at 6 months after sham-irradiation (A) and at 6 months after 18 Gy local heart irradiation (B). 20× Magnification, scale bar: 100 µm.
(TIF)

Figure S3 **Representative micrographs of histological findings in the radiation enteropathy model.** Hematoxylin & Eosin staining at 12 weeks after sham-irradiation (A) and at 12 weeks after 7×5.6 Gy local intestine irradiation (B). 5× Magnification, scale bar: 400 µm.
(TIF)

Figure S4 **Representative micrographs of collagen immunostainings in the radiation enteropathy model.** Collagen type I immunoreactivity (A and B) and collagen type III immunoreactivity (C and D) at 12 weeks after sham-irradiation (A and C) and at 12 weeks after 7×5.6 Gy local intestine irradiation (B and D). 5× Magnification, scale bar: 400 µm.
(TIF)

Figure S5 **Representative micrographs of MPO immunostaining in the radiation enteropathy model.** MPO immunoreactivity at 12 weeks after sham-irradiation (A) and at 12 weeks after 7×5.6 Gy local intestine irradiation (B). 5× Magnification, scale bar: 400 µm.
(TIF)
